# Two cinnamoyl hydroxamates as potential quorum sensing inhibitors against *Pseudomonas aeruginosa*

**DOI:** 10.3389/fcimb.2024.1424038

**Published:** 2024-08-06

**Authors:** Deng Pan, Hua Wu, Jun-Jian Li, Bo Wang, Ai-Qun Jia

**Affiliations:** ^1^ Hainan General Hospital, Hainan Affiliated Hospital of Hainan Medical University, Haikou, China; ^2^ Key Laboratory of Tropical Biological Resources of Ministry of Education, School of Pharmaceutical Sciences, Hainan University, Haikou, China

**Keywords:** cinnamoyl hydroxamates, *Pseudomonas aeruginosa*, quorum sensing inhibitors, biofilms, gentamicin

## Abstract

**Introduction:**

*Pseudomonas aeruginosa* is a ubiquitous pathogen that causes various infectious diseases through the regulation of quorum sensing (QS). The strategy of interfering with the QS systems of *P. aeruginosa*, coupled with a reduction in the dosage of conventional antibiotics, presents a potential solution to treating infection and mitigating antibiotic resistance. In this study, seven cinnamoyl hydroxamates were synthesized to evaluate their inhibitory effects on QS of *P. aeruginosa*. Among these cinnamic acid derivatives, we found cinnamoyl hydroxamic acid (CHA) and 3-methoxy-cinnamoyl hydroxamic acid (MCHA) were the two most effective candidates. Furtherly, the effect of CHA and MCHA on the production of virulence factors and biofilm of *P. aeruginosa* were evaluated. Ultimately, our study may offer promising potential for treating *P. aeruginosa* infections and reducing its virulence.

**Methods:**

The disc diffusion test were conducted to evaluate inhibitory effects on QS of seven cinnamoyl hydroxamates. The influence of CHA and MCHA on the production of virulence and flagellar motility of *P. aeruginosa* was furtherly explored. Scanning electron microscopy (SEM) experiment were conducted to evaluate the suppression of CHA and MCHA on the formed biofilm of *P. aeruginosa*. RT-qPCR was used to detect rhlI, lasA, lasB, rhlA, rhlB, and oprL genes in *P. aeruginosa*. *In silico* docking study was performed to explore the molecular mechanism of CHA and MCHA. The synergistic effects of CHA with gentamicin were detected on biofilm cell dispersal.

**Result:**

After treatment of CHA or MCHA, the production of multiple virulence factors, including pyocyanin, proteases, rhamnolipid, and siderophore, and swimming and swarming motilities in *P. aeruginosa* were inhibited significantly. And our results showed CHA and MCHA could eliminate the formed biofilm of *P. aeruginosa*. RT-qPCR revealed that CHA and MCHA inhibited the expression of QS related genes in *P. aeruginosa*. Molecular docking indicated that CHA and MCHA primarily inhibited the RhlI/R system in *P. aeruginosa* by competing with the cognate signaling molecule C4-HSL.Additionally, CHA exhibited potent synergistic effects with gentamicin on biofilm cell dispersal.

**Discussion:**

*P. aeruginosa* is one of the most clinically and epidemiologically important bacteria and a primary cause of catheter-related urinary tract infections and ventilator-associated pneumonia. This study aims to explore whether cinnamoyl hydroxamates have inhibitory effects on QS. And our results indicate that CHA and MCHA, as two novel QSIs, offer promising potential for treating P. aeruginosa infections and reducing its virulence.

## 1 Introduction

*Pseudomonas aeruginosa*, a highly opportunistic pathogen, with about an 8% detection rate in most Chinese hospitals, commonly colonizes immunocompromised hosts and mechanically ventilated patients (Liu et al., 2023). As a significant contributor to the high mortality rates associated with nosocomial and ventilator-associated pneumonia, *P. aeruginosa* is recognized as one of the most life-threatening bacteria by the World Health Organization (WHO) (Thi et al., 2020). Despite the use of antibiotics like gentamicin for treating infections caused by *P. aeruginosa*, the rampant misuse and overuse of these drugs are accelerating the global crisis of antimicrobial resistance (Mladenovic-Antic et al., 2016).

Quorum sensing (QS) represents a cell-cell communication mechanism found in bacteria that is mediated by cell density. Once the released extracellular signaling molecules reach a certain threshold, QS is triggered to regulate gene expression and several physiological processes, such as virulence factor synthesis, biofilm formation, and bacterial motility (Lee and Zhang, 2015). Therefore, interfering with bacterial QS systems offers a promising alternative strategy for fighting pathogens by attenuating QS-regulated virulence factors (Defoirdt, 2018). In *P. aeruginosa*, the *las*, *rhl*, and *pqs* systems constitute the three major QS pathways. Regulation of these systems is achieved through the action of two LuxR-type receptors (LasR and RhlR) and one LysR-type receptor (PqsR, also known as MvfR), each governing distinct subsets of virulence. The *las* system, positioned at the top of the QS hierarchy, controls the downstream QS systems in response to bacterial cell density (Soukarieh et al., 2018).

Cinnamoyl hydroxamic acid (CHA) is recognized for its role in hydroxamic acid-based histone deacetylase (HDAC) inhibition, and several cinnamoyl hydroxamates have been identified with anticancer properties (Zagni et al., 2019). Previous research conducted by our team revealed four cinnamic acid derivatives with potential QS inhibitor activities (Cheng et al., 2020; Pan et al., 2022; Zhou et al., 2022), which enlightened us to consider whether cinnamoyl hydroxamates also have inhibitory effects on QS because of the structural similarity and it was also known hydroxamates exhibited a range of bioactive effects (Yan et al., 2023). In this study, seven cinnamoyl hydroxamates were synthesized and their QS inhibitory activities against *P. aeruginosa* were evaluated. Notably, two cinnamoyl hydroxamates (CHA and methoxy-cinnamoyl hydroxamic acid (MCHA)) showed excellent QS inhibitory activity against *P. aeruginosa*. Furthermore, CHA exhibited robust synergistic potential in conjunction with gentamicin in inhibiting QS-regulated biofilm formation by *P. aeruginosa.* Our results confirmed that CHA and MCHA are effective QS inhibitors against *P. aeruginosa*, highlighting their potential in augmenting therapeutic strategies against infections caused by this pathogen.

## 2 Materials and methods

### 2.1 Bacterial strains, culture conditions, and reagents

*P. aeruginosa* PAO1 was a kind gift from Prof. Q. H. Gong (Ocean University of China, China). *Chromobacterium violaceum* ATCC 12472 was purchased from the Guangdong Microbial Culture Collection Center (Guangzhou, China). In general, the bacteria were cultured in Luria-Bertani (LB) broth (Hope Bio-Technology, Qingdao, China) under constant shaking (180 rpm) with aeration. Guided by QS inhibitor screening, seven cinnamoyl hydroxamates were identified and synthesized from cinnamic acid according to previous published methods (Usachova et al., 2010), followed by purification via column chromatography and characterization by ^1^H-NMR spectroscopy (AVANCE NEO 400, Bruker BioSpin, Switzerland). More comprehensive details are provided in the Supporting Information. Prior to subsequent experiments, the seven cinnamoyl hydroxamates (a-g) were dissolved in dimethyl sulfoxide (DMSO) to a stock concentration of 100 mg/mL. Antibiotics (gentamicin, polymyxin B, and aztreonam) were purchased from Solarbio (Beijing, China) and dissolved in deionized water.

### 2.2 Screening of QS inhibitory activity by disc diffusion tests

*C. violaceum* ATCC 12472 was used as the indicator strain for the screening of QS inhibitors (Tang et al., 2020). The bacteria were cultured in LB medium (*C. violaceum* ATCC 12472 at 28°C; *P. aeruginosa* PAO1 at 37°C) with shaking at 180 rpm to the logarithmic growth phase, then diluted at a 1:100 ratio with LB solid medium and poured into Petri dishes (90 mm × 15 mm). Subsequently, sterile round filter papers (6 × 6 mm) impregnated with 4 µL of each cinnamoyl hydroxamate solution (100 mg/mL) were added to the dishes, followed by incubation at 28°C for 24 h, with DMSO used as the negative control.

### 2.3 Minimum inhibitory concentration determination and growth curve analysis

To measure the MICs of CHA, MCHA, gentamicin, polymyxin B, and aztreonam, standard broth microdilution was used according to the guidelines of the Clinical and Laboratory Standards Institute (CLSI, 2021). Briefly, 100 μL of cation-adjusted Mueller-Hinton Broth (MHB, Hope Bio-Technology, Qingdao, China) was added to each well of a 96-well polystyrene plate. Subsequently, CHA (0–1.2 mg/mL), MCHA (0–1.2 mg/mL), and antibiotics (0–64 μg/mL) were serially diluted 2-fold from top to bottom in the wells. Overnight-cultured PAO1 strains were diluted with MHB medium and 100 μL of diluted PAO1 strain was added to the plate to achieve a final bacterial concentration of approximately 5 × 10^5^ colony-forming units (CFU)/mL. The plates were prepared in triplicate, then incubated at 37°C for 24 h. The MICs were determined as the lowest concentration of the test solution that inhibited the visible growth of the tested microorganism (Wiegand et al., 2008).

To assess the cytotoxic effects of CHA and MCHA and determine their appropriate sub-MICs for further study, growth curves of PAO1 were monitored in both the presence and absence of CHA and MCHA at specified concentrations. The experimental methods were similar to those used for MIC determination. Specifically, overnight-cultured PAO1 strains were diluted with MHB medium and placed into a 96-well plate. Solutions of CHA or MCHA at sub-MIC levels were added to the wells. The plates were then incubated at 37°C and growth curves were recorded at 620 nm using a microplate reader (Biotek Epoch2, USA) at 3-h intervals over a 24-h period.

### 2.4 Biofilm elimination assays of CHA and MCHA against PAO1

Overnight-cultured PAO1 strains were diluted with trypticase soy broth (TSB, Hope Bio-Technology, Qingdao, China) and added to a 96-well plate (200 μL per well), followed by incubation at 37°C for 24 h. After incubation, the plate was washed with sterile phosphate-buffered saline (PBS, pH = 7.3, 10 mM) to remove the medium and planktonic bacteria. Solutions of CHA or MCHA in TSB medium (200 μL) at designated concentrations were added to each well, and the plates were incubated at 37°C for 17 h. Finally, the plates were gently washed with PBS and the retained biofilms were quantified using crystal violet assay, with absorbance measured at 570 nm (Merritt et al., 2011).

### 2.5 Scanning electron microscopy

For the SEM experiments, overnight-cultured PAO1 strains were diluted with TSB and added to a 24-well plate (1 mL per well). Sterile coverslips (14 × 14 mm) were placed at the bottom of the plate, followed by culture at 37°C for 24 h. After incubation, the plates were washed with sterile PBS to remove the medium and planktonic bacteria. Solutions of CHA or MCHA in TSB (1 mL) at designated concentrations were added to each well, and the plates were incubated again at 37°C for 17 h. The non-adherent cells were then removed by washing the coverslips with PBS. Residual biofilms were fixed using 2.5% glutaraldehyde in a new 24-well plate at 4°C for 4 h, and the samples were dehydrated by increasing concentrations of ethanol (30%, 50%, 70%, 90%, 95%, and 100%, 10 min each). All samples were allowed to dry before SEM-based visualization (Hitachi, S-3000N, Japan) (Zhou et al., 2018a).

### 2.6 QS-regulated virulence factor assays

The pyocyanin quantification assay was performed as described previously (Essar et al., 1990). Briefly, overnight-cultured PAO1 strains were diluted with PDP medium (2% tryptone, 1% K_2_SO_4_, 0.14% MgCl_2_, and 1% glycerol, Hope Bio-Technology, Qingdao, China) and incubated with CHA or MCHA at sub-MICs at 37°C and 180 rpm for 24 h. The known QS inhibitor hordenine (Hor, 1 mg/mL, dissolved in DMSO) was used as a positive control and DMSO was used as a negative control. After centrifugation (10 000 rpm, 4°C, 10 min), the PAO1 culture supernatant (5 mL) was extracted with 3 mL of chloroform, followed by the addition of 0.2 M HCl (1 mL) to the chloroform layer. The upper aqueous phase was then collected by centrifugation at 11 000 × *g* for 10 min and absorbance was measured at 520 nm using a microplate reader (Biotek Epoch2, USA).

Elastase quantitative analysis was performed following the previous methodology (Zeng et al., 2022), with minor modifications. Briefly, overnight-cultured PAO1 strains were diluted with TSB, and 2 mL of PAO1 cultures was incubated with CHA or MCHA at sub-MICs at 37°C, 180 rpm to the logarithmic growth phase. Hor (1 mg/mL) was used as a positive control and DMSO was used as a negative control. The bacterial cultures were subsequently centrifuged at 11 000 ×*g* for 10 min and the supernatants were collected by filtering through a 0.22-μm nylon filter. The subsequent experimental steps were performed according to the protocols provided with the elastase ELISA kit (Enzyme-linked Biotechnology, Shanghai, China).

Protease quantitative analysis was performed according to previously published research (Prithiviraj et al., 2005), with minor modifications. In brief, overnight-cultured PAO1 strains were diluted with TSB, with 1 mL of the bacterial cultures then transferred to a 12-well polystyrene plate and incubated with CHA or MCHA at sub-MICs at 37°C and 180 rpm to the logarithmic growth phase. Hor (1 mg/mL) was used as a positive control and DMSO was used as a negative control. The bacterial cultures were then centrifuged at 11 000 ×*g* for 10 min and the supernatant was filtered through a 0.22-μm nylon filter. Sterile supernatant (150 μL) was added to 250 μL of 0.3% azocasein (w/v)-Tris/HCl solution (50 mM, pH 7.8). The azocasein-treated solutions were then incubated at 37°C for 3 h, after which the reaction was terminated by the addition of 1.2 mL trichloroacetic acid (10%, w/v) for 20 min. Following centrifugation at 11 000 × *g* for 10 min, the supernatant was added to 1 M equal volume of NaOH. Protease activities were determined by measuring absorbance at 440 nm with a microplate reader after CHA or MCHA treatment (Biotek Epoch2, USA).

The effects of CHA or MCHA on *P. aeruginosa* PAO1 rhamnolipids were analyzed using the concentrated H_2_SO_4_-orcinol approach (Grosso-Becerra et al., 2016). In brief, overnight-cultured PAO1 strains were diluted with protease peptone glucose ammonium salt medium (PPGAS, 20 mM NH_4_C1, 20 mM KCl, 120 mM Tris-HCl, 0.5% glucose, 1% protease peptone, 1.6 mM MgSO_4_, adjusted to pH 7.2) and incubated with CHA or MCHA at sub-MICs at 37°C and 180 rpm for 48 h. Hor (1 mg/mL) was used as a positive control and DMSO was used as a negative control. Subsequently, the bacterial cultures were centrifuged at 5 500 ×*g* for 10 min and the supernatants were collected. The supernatants were extracted twice with ethyl acetate, with the organic layers combined and evaporated to dryness at 25°C. The dry extract was resuspended in 500 µL of sterile distilled water, after which 100 µL of the sample was added to 0.19% orcinol in 53% H_2_SO_4_ (900 µL) and incubated in a water bath at 80°C for 30 min. After cooling to room temperature, the OD_421_ was measured.

Siderophore quantitative analysis was performed as described previously (Yin et al., 2022). Briefly, overnight-cultured PAO1 strains were first diluted with TSB, with 5 mL of these cultures then incubated with CHA or MCHA at sub-MICs at 37°C and 180 rpm to the logarithmic growth phase. Hor (1 mg/mL) was used as a positive control and DMSO was used as a negative control. The bacterial cultures were then centrifuged at 11 000 ×*g* for 10 min and the supernatants were collected using a 0.22-μm nylon filter. Subsequently, 3 mL of filtrate was mixed with an equal volume of chrome azurol S (CAS) test solution (Coolaber, Beijing, China), and incubated for 1 h in the dark at room temperature. After incubation, reaction solution absorbance was determined at 680 nm using a microplate reader (Biotek Epoch2, USA).

### 2.7 Flagellar motility assay of CHA or MCHA against *P. aeruginosa*

Swimming and swarming motilities were assessed as described previously (Rashid and Kornberg, 2000). Briefly, 2 µL of PAO1 grown in LB for 17 h was diluted in fresh sterile LB medium to obtain an OD_620_ of 0.5, then spotted onto the center of swimming plates (0.8% nutrient broth, 0.5% glucose, 0.3% agar) or swarming plates (0.8% nutrient broth, 0.5% glucose, 0.5% agar). The plates were supplemented with CHA or MCHA or not. After 17–24 h of static incubation at 37°C, swimming and swarming motilities were directly observed at the air-agar interface.

### 2.8 Real-time quantitative PCR

Overnight-cultured PAO1 strains diluted in LB medium with CHA (200 μg/mL) or MCHA (300 μg/mL) were incubated at 37°C and 180 rpm for 17 h. After washing the biofilm cells with PBS, the attached cells were scraped and collected in a microtube. Total RNA was extracted using a UNIQ-10 column Trizol total RNA extraction kit (Sangon Biotech, Shanghai, China) and extracted RNA purity was detected by electrophoresis. The samples were then reverse-transcribed to single-stranded complementary DNA (cDNA) in a 20-µL volume of the reaction mixture using Maxima Reverse Transcriptase (200 U, Thermo Fisher Scientific, Waltham, MA, USA). Then, RT-qPCR was performed in a 20-µL reaction volume, containing 10 µL of SYBR Green qPCR Master Mix (Sangon Biotech, Shanghai, China), 2 µL of template cDNA, 0.8 µL of each primer (Sangon Biotech, Shanghai, China), and 7.2 µL of DNase/RNase-free water. After that, 96-well plates with added samples were placed in an ABI Stepone plus fluorescence PCR instrument (ABI, Foster City, USA) for reaction. The qPCR conditions were 95°C for 3 min, followed by 45 cycles of 95°C for 5 s and 60°C for 30 s. Amplification of specific transcripts was confirmed by melting curve profiles generated at the end of the PCR program. Fold changes in expression versus control conditions were determined using the 2^−ΔΔCt^ method, with *rpsL* as a housekeeping gene (Grosso-Becerra et al., 2016) (see **Supplementary Table S1** for primer sequences).

### 2.9 *In silico* docking study

Co-crystallized structures of LasR (PDB code: 2UV0) and PqsR (PDB code: 4JVD) were obtained from the Protein Data Bank (http://www.rcsb.org/pdb/) (Berman et al., 2000). Another structure of the homology RhlR model was downloaded from supporting information in a previously reported reference (Nam et al., 2020). As the crystal structure of LasR was a tetramer in 2UV0, one monomer was selected for docking calculation. Schrödinger software release 2017-1 (New York, USA) was used to carry out *in silico* calculations. Before docking calculations, several necessary structural preparations of the protein were required, including removal of dispensable water molecule removal, hydrogen addition, and minimization. All 3D ligand structures were prepared using the LigPrep module (LigPrep, Schrödinger, New York, USA), optimized through the OPLS3 force field (Harder et al., 2016). Glide XP (extra precision) in the Schrodinger suite was used for ligand docking. The binding energy of each ligand was calculated through the OPLS3 force field. Finally, the docking pose was optimized while the protein was kept fixed.

### 2.10 Checkerboard assays

The interactions between CHA or MCHA and antibiotics (gentamicin, polymyxin B, aztreonam) were examined against the *P. aeruginosa* PAO1 strain using the broth microdilution checkerboard assay, with the fractional inhibitory concentration index (FICI) used to analyze the types of interactions between compounds (Ham et al., 2021). Briefly, CHA or MCHA and one antibiotic were diluted with MHB medium into a series of concentrations based on the MICs of CHA and MCHA. Similarly, overnight-grown bacterial colonies were also diluted with MHB to a final concentration of approximately 5 × 10^5^ CFU/mL in each well. Results were observed after static incubation at 37°C for 24 h. All experiments were performed in triplicate. The FICIs were calculated using the equation: FICI = MIC (antibiotic combined with CHA or MCHA)/MIC (antibiotic alone) + MIC (CHA or MCHA combined with antibiotic)/MIC (CHA or MCHA alone), where FICI ≤ 0.5, 0.5 < FICI ≤ 4, and FICI > 4 mean synergistic, indifferent, and antagonistic effects, respectively (Zhou et al., 2018b).

### 2.11 Effects of CHA combined with gentamicin on PAO1 biofilm elimination

#### 2.11.1 Crystal violet staining analysis

The methods employed were the same as those used for the biofilm elimination assay (Section 2.4). This experiment was performed with four distinct groups: DMSO negative control, 50 μg/mL CHA, 2 μg/mL gentamicin, and CHA (50 μg/mL) combined with gentamicin (2 μg/mL).

#### 2.11.2 Quantification of CFU assay

Biofilms were grown as described in Section 2.4. Following a 24 h incubation period, plates were washed with sterile PBS to remove any remaining medium and planktonic bacteria. Subsequently, fresh sterilized TSB medium was used for dilution, to which was added either 50 μg/mL CHA, 2 μg/mL gentamicin, or a combination of both to a final volume of 200 μL per well, followed by incubation at 37°C for 17 h. DMSO was used as a negative control. After that, biofilm cells were detached by sonication at 300 W power and 40 kHz for 5 min. Sterile PBS was added, followed by vigorous mixing. The resulting solution was then serially diluted in a 10-fold gradient and plated onto LB agar plates. Colonies were counted and the CFU per biofilms were calculated.

### 2.12 SEM analysis

The procedures used to assess the morphology of biofilms after their elimination followed those described in Section 2.5, except for the division into four groups: DMSO as the negative control, 50 μg/mL CHA, 2 μg/mL gentamicin, and the combination of CHA (50 μg/mL) with gentamicin (2 μg/mL).

### 2.13 Statistical analysis

Data are presented as mean ± standard deviation (SD). Experiments were performed in triplicate and statistical analyses were executed using one-way analysis of variance (ANOVA) with Dunnett test or Student’s *t*-test. Statistically significant values were defined as *P* < 0.05, < 0.01, or < 0.001. GraphPad Prism v8 (GraphPad Software Inc., San Diego, CA, USA) was used for all graphical and statistical evaluations.

## 3 Results

### 3.1 Assessment of QS inhibition by CHA and MCHA

**Figure 1:**
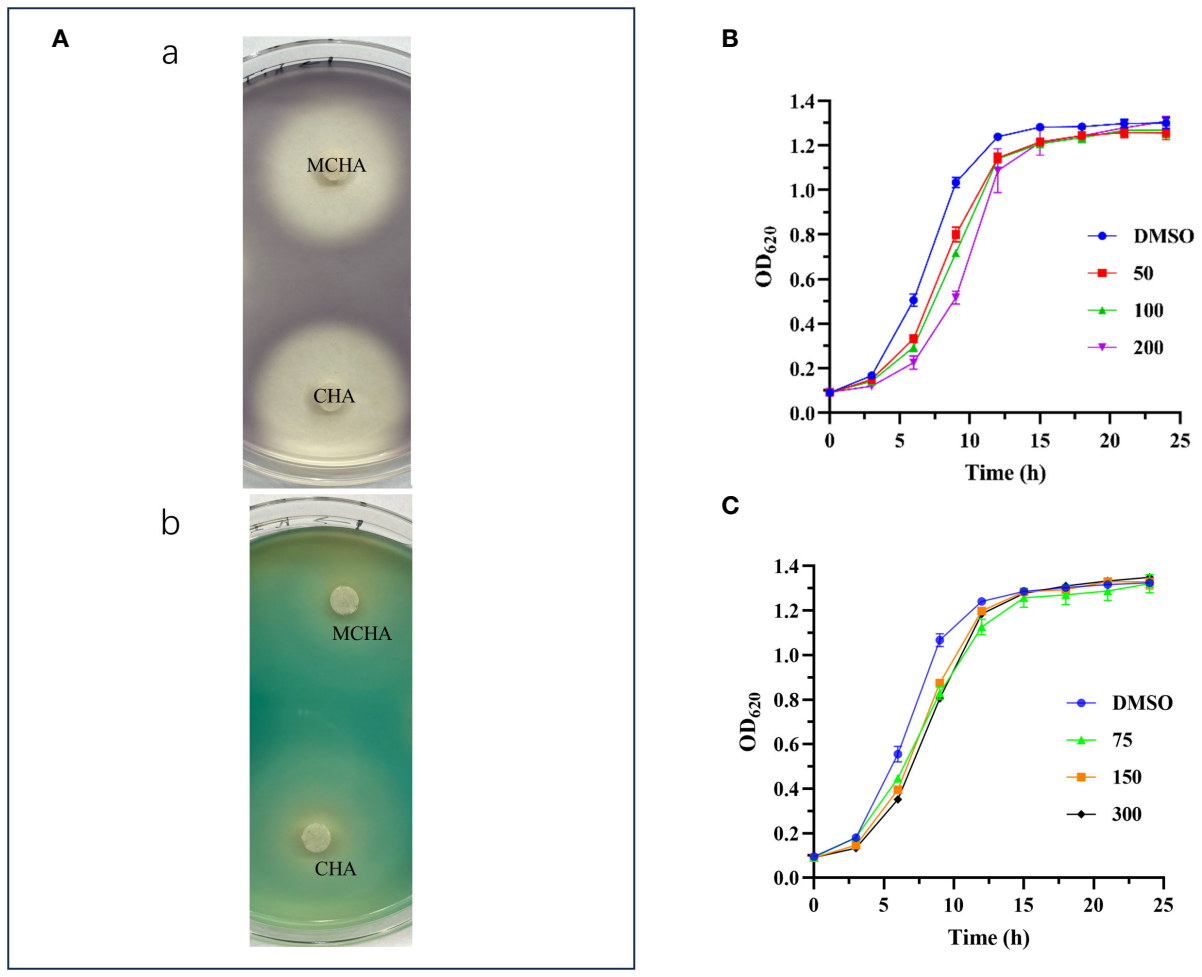
QS inhibitory activities of CHA and MCHA. **(A)** QS inhibitory activities of CHA and MCHA on a Petri dish against strains *C. violaceum* ATCC 12472 (a) and *P. aeruginosa* PAO1 (b). Growth curves of *P. aeruginosa* PAO1 treated with CHA **(B)** and MCHA **(C)** at sub-MICs, respectively.

The production of purple pigment (violacein) in *C. violaceum* ATCC 12472 is regulated by the QS systems. This result is a naturally occurring and readily observable phenotype, making it a commonly used indicator strain for QSI (McLean et al., 2004). In the present study, as illustrated in **Figure 1A**, the presence of CHA or MCHA resulted in the formation of a white and turbid zone around the filter paper discs, signifying a notable suppression of synthesis of both violacein in *C. violaceum* ATCC 12472 and pyocyanin in *P. aeruginosa* PAO1. In contrast, the other five cinnamoyl hydroxamates (c-g) exhibited minimal inhibitory effects (see **Supporting Information**). These findings confirm the QS inhibitory potential of both CHA and MCHA.

### 3.2 Susceptibility of PAO1 to CHA, MCHA, and antibiotics

The MICs of CHA, MCHA, gentamicin, polymyxin B, and aztreonam against *P. aeruginosa* PAO1, assessed using a broth microdilution assay, were 400 μg/mL, 600 μg/mL, 4 μg/mL, 4 μg/mL, and 4 μg/mL, respectively. Growth curve analysis indicated that CHA (50–200 µg/mL) and MCHA (75–300 µg/mL) did not inhibit *P. aeruginosa* growth (**Figures 1B, C**), although the growth rate of *P. aeruginosa* was slower than that in control group in the initial period. Subsequent experiments utilized sub-MICs for CHA (low-dose group (L-CHA): 50 µg/mL; middle-dose group (M-CHA): 100 µg/mL; high-dose group (H-CHA): 200 µg/mL) and MCHA (low-dose group (L-MCHA): 75 µg/mL; middle-dose group (M-MCHA): 150 µg/mL; high-dose group (H-MCHA): 300 µg/mL). The FICIs of all combinations exceeded 0.5, indicating that all compounds showed no synergistic or antagonistic interaction with the tested antibiotics against PAO1 planktonic cells (**Supplementary Table S2**).

### 3.3 Effects of CHA and MCHA on biofilms

**Figure 2:**
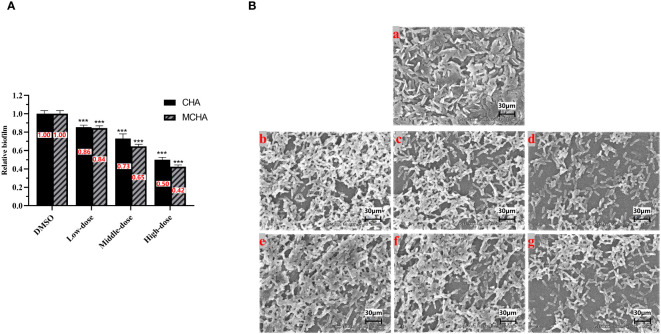
Effects of CHA and MCHA on *P. aeruginosa* PAO1 biofilm disruption. **(A)** Analysis of crystal violet staining assay with CHA and MCHA; **(B)** SEM images of formed *P. aeruginosa* biofilms treated with (a) DMSO, (b) 50, (c) 100, (d) 200 µg/mL CHA, or (e) 75, (f) 150, (g) 300 µg/mL MCHA, respectively. Data are presented as absorbance of mean ± SD of three independent experiments. ***P < 0.001 compared to DMSO control group by one-way ANOVA, n = 3. Low-dose group (CHA: 50 µg/mL; MCHA: 75 µg/mL); Middle-dose group (CHA: 100 µg/mL; MCHA: 150 µg/mL); High-dose group (CHA:200 µg/mL; MCHA: 300 µg/mL).

The effects of CHA and MCHA on *P. aeruginosa* PAO1 formed biofilms in 96-well plates were evaluated using crystal violet staining. As shown in **Figure 2A**, *P. aeruginosa* PAO1 formed biofilms were significantly eliminated by CHA and MCHA in a dose-dependent manner. Compared to the control group, the reduction in formed biofilms reached 50% and 58% under CHA (200 μg/mL) and MCHA (300 μg/mL) treatment, respectively. SEM analysis revealed CHA and MCHA treatments transformed *P. aeruginosa* PAO1 biofilms from dense to more porous and less compact structures, in line with crystal violet staining results, demonstrating concentration-dependent effects, while also obviously reducing extracellular secretion and bacterial aggregation (**Figure 2B**). These findings demonstrate that exposure to CHA and MCHA can significantly disrupt formed biofilms of *P. aeruginosa* PAO1.

### 3.4 Effects of CHA and MCHA on *P. aeruginosa* PAO1 virulence factors

**Figure 3:**
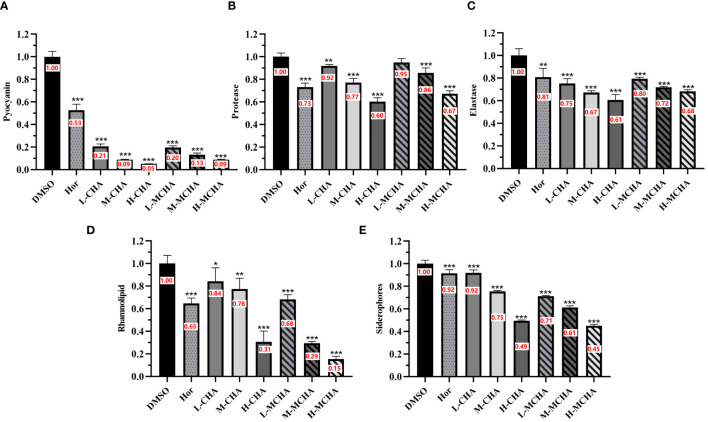
Inhibitory effects of CHA and MCHA on *P. aeruginosa* PAO1 virulence factors. **(A)** Pyocyanin; **(B)** Protease; **(C)** Elastase; **(D)** Rhamnolipid; **(E)** Siderophores Data are presented as absorbance of mean ± SD of three independent experiments. *P < 0.05, **P < 0.01, ***P < 0.001 compared to DMSO control group by one-way ANOVA, n = 3. L-CHA: CHA low-dose group (50 µg/mL); M-CHA: CHA middle-dose group (100 µg/mL); H-CHA: CHA high-dose group (200 µg/mL); L-MCHA: MCHA low-dose group (75 µg/mL); M-MCHA: MCHA middle-dose group (150 µg/mL); H-MCHA: MCHA high-dose group (300 µg/mL).

The effects of CHA and MCHA on pyocyanin production by *P. aeruginosa* PAO1 were first examined. As shown in **Figure 3A**, CHA and MCHA exerted significant inhibitory effects on pyocyanin production. Compared to the control group, CHA (200 μg/mL) and MCHA (300 μg/mL) inhibited pyocyanin production by 95% and 92%, respectively, both exceeding that of the positive control Hor (47%).

Subsequently, the effects of CHA and MCHA on elastase and protease activities in *P. aeruginosa* PAO1 were determined. As shown in **Figure 3B**, elastase activity was inhibited by CHA and MCHA in a concentration-dependent manner. CHA at 50 μg/mL, 100 μg/mL, and 200 μg/mL achieved elastase inhibition rates of 25%, 33%, and 39%, respectively, while MCHA at 75 μg/mL, 150 μg/mL, and 300 μg/mL achieved elastase inhibition rates of 21%, 28%, and 32%, respectively, exceeding that of the positive control Hor (19%). In addition, while CHA and MCHA did not significantly affect protease activity at lower concentrations (**Figure 3C**), treatment with CHA at 200 μg/mL and MCHA at 300 μg/mL inhibited protease activity by 40% and 33%, respectively, surpassing that of Hor (27%).

The effects of CHA and MCHA on the production of rhamnolipid by *P. aeruginosa* PAO1 were also evaluated. As shown in **Figure 3D**, CHA at 200 μg/mL and MCHA at 300 μg/mL inhibited rhamnolipid production by 69% and 85%, respectively, both exceeding the inhibitory effects of Hor (35%).

The CAS assay, composed of chrome azurol sulphonate, hexadecyl-trimethyl-ammonium bromide (HDTMA), and iron ions, changes from blue to orange-yellow when siderophores from *P. aeruginosa* bind to iron ions, enabling siderophore detection. As shown in **Figure 3E**, CHA and MCHA significantly reduced siderophore production, with CHA at 200 μg/mL and MCHA at 300 μg/mL inhibiting siderophore activity by 51% and 55%, respectively, surpassing the 8% inhibition achieved by Hor.

### 3.5 Effects of CHA and MCHA on *P. aeruginosa* PAO1 motility

**Figure 4:**
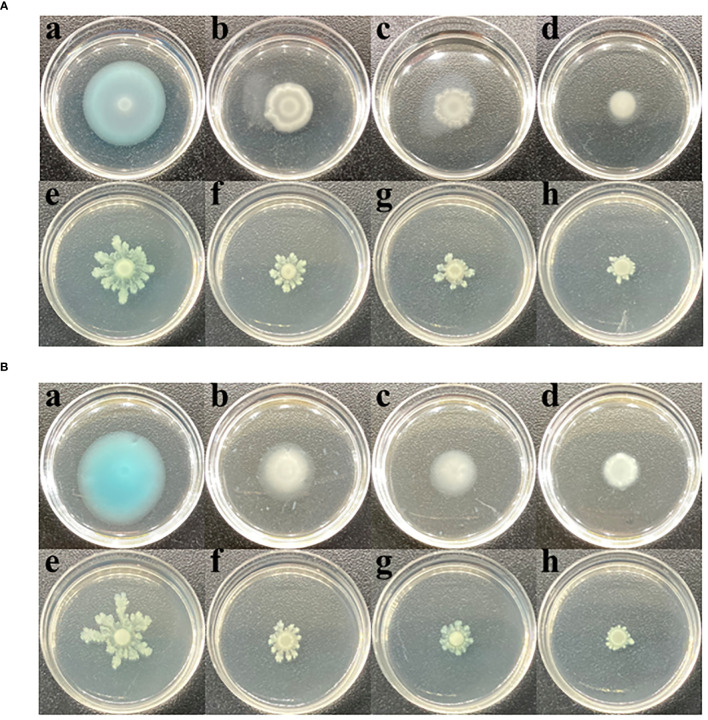
Effects of CHA and MCHA on swimming and swarming motilities of *P. aeruginosa* PAO1 at sub-MICs. **(A)** The effects of CHA on swimming (a. DMSO; b. CHA 50 µg/mL; c. CHA 100 µg/mL, d. CHA 200 µg/mL) and swarming (e. DMSO; f. CHA 50 µg/mL; g. CHA 100 µg/mL, h. CHA 200 µg/mL). **(B)** The effects of MCHA on swimming (a. DMSO; b. CHA 75 µg/mL; c. CHA 150 µg/mL, d. CHA 300 µg/mL) and swarming (e. DMSO; f. CHA 75 µg/mL; g. CHA 150 µg/mL, h. CHA 300 µg/mL).

Enhanced motility facilitates bacterial infection of hosts. This study explored the effects of CHA and MCHA on *P. aeruginosa* PAO1 motility via swimming and swarming experiments. As shown in **Figure 4**, after treatment sub-MICs of CHA and MCHA, both the swimming and swarming motilities of *P. aeruginosa* PAO1 were significantly suppressed. The diameter of the swimming diffusion zone decreased from 3.1 ± 0.2 cm in the untreated DMSO group to 1.8 ± 0.1 cm, 1.4 ± 0.1 cm, and 0.9 ± 0.1 cm in the 50 μg/mL, 100 μg/mL, and 200 μg/mL CHA-treated groups and to 1.9 ± 0.1 cm, 1.5 ± 0.1 cm, and 1.1 ± 0.1 cm in the 75 μg/mL, 150 μg/mL, and 300 μg/mL MCHA-treated groups, respectively.

### 3.6 Effects of CHA and MCHA on QS-related gene expression

**Figure 5:**
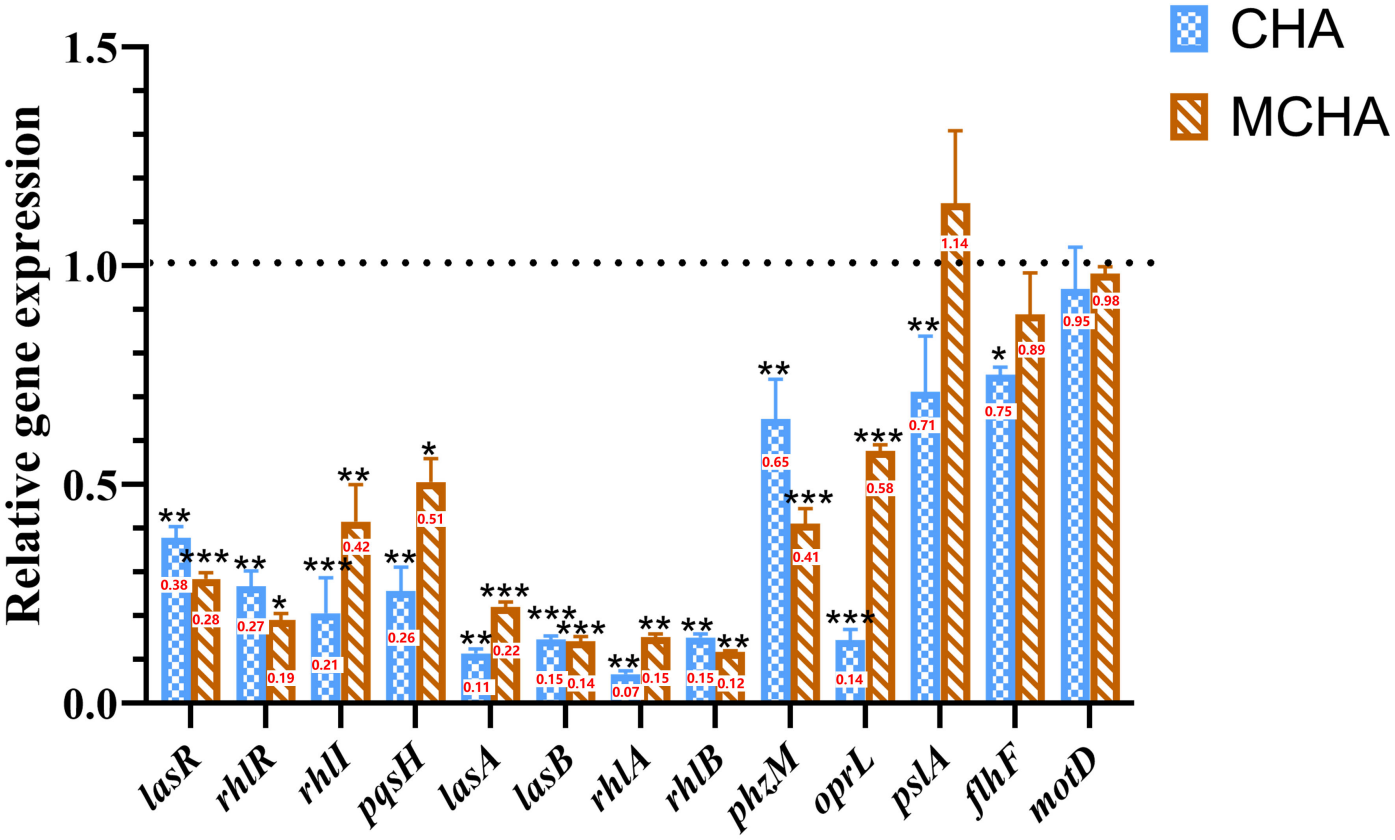
Effects of CHA and MCHA on QS and virulence-related genes in *P. aeruginosa* PAO1. Data are averages of the three experiments, expressed as multiple ± SD of gene expression level, n = 3. Data are presented as expression fold-changes of mean ± SD of three independent experiments. *P < 0.05, **P < 0.01, ***P < 0.001 compared to DMSO control group in *t*-test, n = 3.

To further investigate the mechanism by which CHA and MCHA inhibit the QS system, biofilm formation, and motility of *P. aeruginosa* PAO1, RT-qPCR was used to examine the gene expression changes. As shown in **Figure 5**, CHA (200 μg/mL) and MCHA (300 μg/mL) treatments reduced the expression of PAO1 QS-related genes to varying degrees. Notably, *lasR*, which encodes the transcriptional regulator protein LasR in the LasI/LasR system, was down-regulated by 62% (CHA) and 72% (MCHA); *rhl*R, encoding the transcriptional regulator protein RhlR in the RhlI/R system, was down-regulated by 73% (CHA) and 81% (MCHA); *rhl*I, a butyl-homoserine lactone (C4-HSL) synthase gene, was down-regulated by 80% (CHA) and 58% (MCHA); and *pqs*H, a 2-heptyl-3-hydroxy-4(1H)-quinolone synthase gene in the *pqs* system, was downregulated by 75% (CHA) and 50% (MCHA). Significant down-regulation was also observed in *P. aeruginosa* PAO1 virulence factor-related genes, with *lasA*, encoding protease LasA synthesis, inhibited by 89% (CHA) and 78% (MCHA); *lasB*, encoding elastase LasB synthesis, inhibited by 86% (CHA and MCHA); *rhl*A and *rhl*B, rhamnolipid synthesis-related genes, inhibited by 93% and 85% (CHA) and by 85% and 88% (MCHA), respectively; and *phz*M, a pyocyanin synthesis-related gene, inhibited by 36% (CHA) and 59% (MCHA). Results also showed that CHA and MCHA exerted inhibitory effects on biofilm and motility-related genes, with *opr*L, encoding peptidoglycan-associated lipoprotein synthesis, inhibited by 86% (CHA) and 43% (MCHA); *psl*A, encoding biofilm formation proteins, inhibited by 30% (CHA); and *flh*F and *mot*D, encoding the flagellar biosynthesis regulator and flagellar motor protein, down-regulated by 25% (CHA) and 6% (CHA), respectively.

### 3.7 Docking simulations

**Figure 6:**
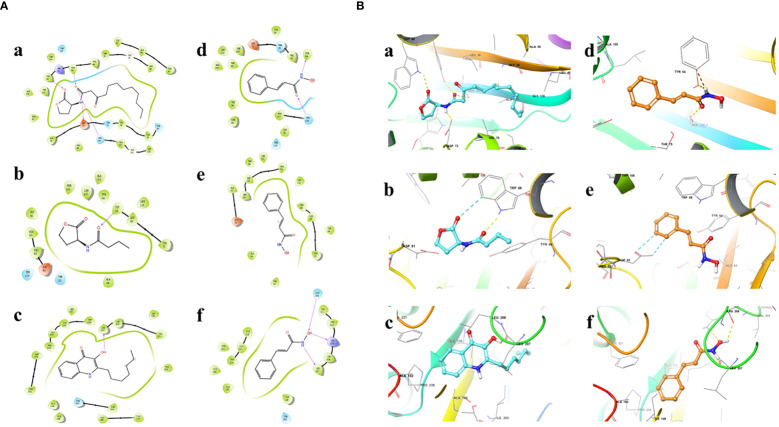
2D **(A)** and 3D **(B)** docking diagrams of receptor proteins LasR (a, d), RhlR (b, e), and PqsR (c, f) with 3-oxo-C12-HSL (a), C4-HSL (b), PQS (c), and CHA (d–f).

**Figure 7:**
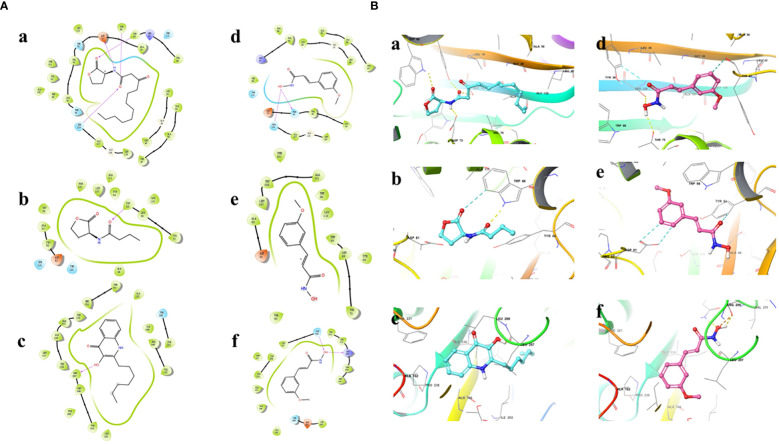
2D **(A)** and 3D **(B)** docking diagrams of receptor proteins LasR (a, d), RhlR (b, e) and PqsR (c, f) with 3-oxo-C12-HSL (a), C4-HSL (b), PQS (c), and MCHA (d–f).

Results showed that the interactions of CHA and MCHA with the LasR and PqsR proteins were weaker than those with their ligands N-3-oxododecanoyl homoserine lacton (3-oxo-C12-HSL) and 2-heptyl-3,4-dihydroxyquinoline (PQS, quinolone signal molecule), respectively (**Figures 6**, **7** and **Table 1**). Conversely, the binding energies of CHA and MCHA with the RhlR was -31.19, -31.58 kcal/mol, respectively. Both of them were lower than -26.80 kcal/mol, the binding energy of the signal molecule (C4-HSL) with the RhlR. This result indicated that the docking of CHA and MCHA with the RhlR protein was stronger than that of the C4-HSL ligand. These findings suggest that CHA and MCHA predominantly target the RhlI/R system to inhibit QS in *P. aeruginosa*, consistent with the RT-qPCR results.

**Table 1:** Binding energy of autoinducers, CHA, and MCHA to their target receptors and amino acid residues involved in their complex formation.

Receptors	Ligands	Hydrogen bonds	Aromatic H-Bond	Docking scores	ΔG(bind) kcal/mol
LasR	3-oxo-C12-HSL	Tyr56, Trp60, Asp73, Ser129	–	-9.631	-90.21
LasR	CHA	Tyr64, Ser129	–	-6.646	-42.47
LasR	MCHA	Thr75, Ser129	Tyr47, Tyr56	-6.906	-52.24
RhlR	C4-HSL	Trp68	Trp68	-5.327	-26.80
RhlR	CHA	–	Asp81	-6.426	-31.19
RhlR	MCHA	–	Asp81, Tyr64	-6.564	-31.58
PqsR	PQS	Leu207	–	-7.735	-51.38
PqsR	CHA	Gln194, Leu207, Arg209	–	-5.837	-35.89
PqsR	MCHA	Gln194, Arg209	–	-6.832	-36.86

### 3.8 Synergistic anti-biofilm activity of CHA in combination with gentamicin

**Figure 8:**
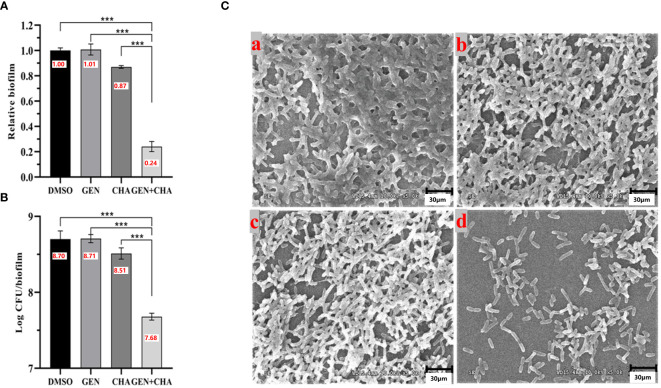
Destruction effects of CHA (50 μg/mL) and gentamicin (2 μg/mL) alone and in combination on *P. aeruginosa* PAO1 biofilms. **(A)** Crystal violet staining; **(B)** Log CFU/biofilms; **(C)** SEM images of formed biofilms of *P. aeruginosa* PAO1 treated with (a) DMSO, (b) 2 µg/mL gentamicin, (c) 50 µg/mL CHA, or (d) 2 µg/mL gentamicin and 50 µg/mL CHA, respectively. Data are presented as absorbance of mean ± SD of three independent experiments. ***P < 0.001 versus corresponding control by one-way ANOVA, n = 3.

Due to its lowest FICI value, gentamicin was selected to investigate its potential synergistic anti-biofilm effects with CHA on *P. aeruginosa* PAO1. Given that the optimal blood concentration of gentamicin is 4–8 μg/mL, and its MIC against *P. aeruginosa* PAO1 was determined to be 4 μg/mL, half the optimal blood concentration (2 μg/mL) was used for subsequent experiments. Biofilm formation significantly contributes to pathogenic resistance. Here, crystal violet staining (**Figure 8A**) revealed that 2 μg/mL gentamicin alone had negligible impact on *P. aeruginosa* PAO1 biofilm formation, while 50 μg/mL CHA independently reduced biofilm formation by 13%. Notably, when the same concentration of gentamicin (2 μg/mL) and CHA (50 μg/mL) were used together, the *P. aeruginosa* PAO1 biofilm clearance rate reached 76%. Furthermore, CFU analysis obtained similar results (**Figure 8B**), showing a 90.7% reduction in viable bacteria in the biofilms, suggesting that CHA enhanced the anti-biofilm effects of gentamicin on *P. aeruginosa* PAO1. Consistently, SEM analysis (**Figure 8C**) illustrated that the DMSO group biofilms were dense and thick, with the 2 μg/mL gentamicin and 50 μg/mL CHA treatments alone showing no significant effect on biofilm clearance. In contrast, the combined gentamicin and CHA group showed a notable reduction in biofilms thinness and strength, allowing gentamicin more easily to penetrate and kill the bacteria, thus restoring the sensitivity of *P. aeruginosa* PAO1 to gentamicin.

## 4 Discussion

*P. aeruginosa* is one of the most clinically and epidemiologically important bacteria and a primary cause of catheter-related urinary tract infections and ventilator-associated pneumonia (Tenover et al., 2022). Of concern, its resistance to multiple antibiotics, such as tazobactam, carbapenems, fluoroquinolones, ceftazidime, aminoglycosides, and polymyxins, is increasing (de Lacerda Coriolano et al., 2021). Consequently, targeting QS has emerged as a new promising antibacterial strategy, which is capable of preventing the development of bacterial resistance and suppressing virulence factor production. In our previous works, we found four cinnamic acid derivatives exhibited inhibitory activities on QS (Cheng et al., 2020; Pan et al., 2022; Zhou et al., 2022). In this study, according to the drug-likeness analysis, a total of seven cinnamoyl hydroxamates were synthesized selectively. After QSI screenings against test strain ATCC 12472 and PAO1, CHA and MCHA showed excellent QS inhibitory effects against ATCC 12472 and PAO1, among which CHA showed more potent than MCHA bearing 3-methoxy. Unfortunately, four cinnamoyl hydroxamates bearing methoxy or hydroxy at *ortho*- or *para*- position of phenyl showed weak or no QSI activities. Subsequently, two cinnamoyl hydroxamates bearing fluoro groups also showed no QSI activities against ATCC 12472 and PAO1.

Based on the above findings, we investigated the inhibitory effects of CHA and MCHA on the virulence factor production, motility, and biofilm formation in *P. aeruginosa* PAO1 at sub-MICs. The mechanisms by which CHA and MCHA impact QS systems were initially explored by detecting the mRNA levels of QS-related genes and conducting molecular docking analysis. Furthermore, the potential synergistic effects of combining CHA with gentamicin against PAO1 were investigated.

Many extracellular virulence factors in *P. aeruginosa*, such as pyocyanin, proteases, rhamnolipid, and siderophore, play crucial roles in its pathogenicity, invasive ability, and toxicity (Strateva and Mitov, 2011). Pyocyanin, a blue redox-active secondary metabolite regulated by the *rhl* QS system, inactivates catalase and modulates *L-glutathione* (GSH) redox cycling in lung cells. *In vivo* experiments have also indicated that the bacterial load of pyocyanin-deficient mutant strains is 1 000–10 000 lower than that of their respective wild-type counterparts (Lau et al., 2004; Morkunas et al., 2012). *P. aeruginosa* secretes multiple proteases, including elastase, which are under QS regulation and essential for survival within hosts. These proteases can damage host tissue and degrade host immunoglobulins (Pearson et al., 1997; Li and Lee, 2019; Aldawsari et al., 2021). Rhamnolipids, surfactant-active virulence factors in *P. aeruginosa*, facilitate immune cell and erythrocyte destruction, swarming and twitching motility, and biofilm formation (Laabei et al., 2014), with their production dependent on both the *rhl* system and *rhlAB* (encoding a rhamnosyltransferase) (Pearson et al., 1997). Siderophores are vital for acquiring iron, crucial for processes such as electron transport and DNA replication, and serve as key virulence factors in *P. aeruginosa*, significantly affecting biofilm formation and virulence (Jeong et al., 2023). Popat et al. (2017) reported that two major siderophores in *P. aeruginosa*, pyoverdine and pyochelin, are regulated by the *P. aeruginosa* QS quinolone signal molecule (PQS). Therefore, targeting virulence factor production offers a viable approach to combat *P. aeruginosa* infections. In this study, our results demonstrated that CHA and MCHA significantly inhibited pyocyanin, protease, elastase, rhamnolipid, and siderophore production, outperforming the efficacy of the positive control Hor. Notably, compared to the negative control, pyocyanin production was reduced to 5% after treatment with 200 μg/mL CHA or MCHA. These findings were supported by RT-qPCR analysis, showing significant down-regulation in the expression of genes regulating virulence factors (Ullah et al., 2017; Ahmed et al., 2019; Yang et al., 2021), including *phz*M (pyocyanin), *las*A (proteases), *las*B (elastase), *rhl*A/B (rhamnolipid), and *opr*l (outer membrane proteins) in *P. aeruginosa* PAO1 post-treatment with CHA or MCHA. These outcomes suggest that CHA and MCHA hold promise in attenuating the pathogenicity of *P. aeruginosa.*

Swimming and swarming motilities, regulated by the *las* and *rhl* QS systems in *P. aeruginosa* (Köhler et al., 2000; Sonbol et al., 2022), are important virulence traits. Swimming motility, driven by a single polar flagellum, is crucial for locating infection sites, while swarming motility, which relies on multiple flagella, plays an essential role in the early stage of biofilm establishment (Khan et al., 2020). In the current study, treatment with CHA and MCHA significantly reduced swimming and swarming motilities in *P. aeruginosa* PAO1 compared to the control group, suggesting that these compounds can effectively inhibit *P. aeruginosa* motility. These inhibitory effects were further confirmed by RT-qPCR analysis, which showed a decrease in the expression of motility-related genes *flh*F and *mot*D (Lee et al., 2011). Biofilms, complex structures composed of autogenic extracellular polymeric substances such as polysaccharides, extracellular DNA, proteins, and lipids, enable bacteria to survive adverse conditions, including antibiotic exposure and host immune responses (Vestby et al., 2020). Studies have shown that bacteria within biofilms exhibit resistance up to 1000 times greater than their planktonic counterparts. Known for its proficiency in forming biofilms, *P. aeruginosa* poses significant challenges in clinical environments due to this robust defense mechanism (Thi et al., 2020). In the present research, we found that both CHA and MCHA effectively eliminated formed biofilms in *P. aeruginosa*, by disrupting biofilm architecture, which leaded to a more dispersed and less cohesive structure. Additionally, treatment with CHA significantly reduced the expression of the *psl*A gene, which regulates the production of exopolysaccharides to provide structural support during the primary stage of biofilm formation (Colvin et al., 2012). The observed inhibitory effects on motility and biofilm formation suggest that CHA and MCHA may limit *P. aeruginosa* proliferation and reduce antibiotic resistance, highlighting their potential as candidate agents in treating *P. aeruginosa* infections.

QS is a communication system employed by bacterial populations to coordinate virulence gene expression, representing a promising new target for antimicrobial chemotherapy (Vattem et al., 2007). The pathogenicity of *P. aeruginosa* is mediated through its QS systems, notably the *las*, *rhl*, and *pqs* systems. Here, we explored the effects of CHA and MCHA on QS-regulated genes in *P. aeruginosa* PAO1, with RT-qPCR analysis revealing a marked down-regulation in the expression of QS-regulated genes *las*R, *rhl*R, *rhl*I, and *pqs*H following treatment. Among the QS systems, the *las* system is pivotal, orchestrating the activation of the *rhl* and *pqs* systems (Chadha et al., 2022). Notably, CHA and MCHA both induced the down-regulation of *lasR*. Ahmed et al. (2019) showed that *trans*-cinnamaldehyde effectively down-regulates *lasI* and *lasR* levels in *P. aeruginosa*, although the exact mechanism remains unclear. Maura et al. (2016) reported that PqsR controls *lasR* and *rhlR* expression during the early phases of bacterial growth, while RhlR suppresses (PqsR) the QS system during the late exponential and stationary phases. Ueda et al. (2009) also found that uracil can influence all three QS pathways in *P. aeruginosa*. We suspect that CHA and MCHA may influence the autoregulatory loops of the three QS systems or the uridine monophosphate (UMP) synthesis pathway, thereby reducing *lasR* levels. However, the specific mechanism needs to be explored in further studies.

Molecular docking calculations were performed to investigate the possible interactions between CHA or MCHA and receptors of QS signaling molecules in *P. aeruginosa* PAO1. The QS signaling molecules 3-oxo-C12-HSL, C4-HSL, and PQS, were docked against the LasR, RhlR, and PqsR receptors respectively (Kumar et al., 2015). The *in silico* molecular docking results demonstrated that both CHA and MCHA could bind to LasR, RhlR, and PqsR, achieving high ligand-receptor docking scores with all three receptors. Notably, the CHA-RhlR and MCHA-RhlR complexes showed the highest docking scores, surpassing that of the natural autoinducer C4-HSL. Even though only aromatic and weaker H-bond interactions but no hydrogen bond was detected between CHA or MCHA with RhlR in *silico* docking analysis. We speculate the great matching degree between CHA or MCHA and RhlR might contribute lower binding energy between them. On the whole, these findings suggest that CHA and MCHA have the potential to act as potent inhibitors of LasR, RhlR, and PqsR, with particularly pronounced inhibitory effects against RhlR, potentially functioning as antagonists to these receptor signals.

Combinatorial therapies have garnered increasing attention due to their ability to target multiple therapeutic pathways (Vasudevan et al., 2018). Numerous QSIs have shown synergistic effects with antibiotics. For instance, Balaban et al. (2003) demonstrated that combining the RNAIII-inhibiting peptide (RIP) with conventional antibiotics completely eradicated *Staphylococcus epidermidis* infections *in vivo*. Roudashti et al. (2017) reported enhanced effectiveness of curcumin in combination with ceftazidime and ciprofloxacin against the QS system in *P. aeruginosa*. In our previous research, we highlighted the synergistic effects of various QSIs (Zhou et al., 2018b; Cheng et al., 2020; Pan et al., 2022; Xu et al., 2022). In this study, we found gentamicin (2 μg/mL) exhibited little effect on biofilms of *P. aeruginosa*, while CHA-alone (50 μg/mL) treatment only resulted in a slight decrease of biofilms. Nevertheless, it was notable that combinatorial administration of CHA (50 μg/mL) with gentamicin (2 μg/mL) significantly reduced biofilm formation and increased biofilm cell dispersal compared to individual treatments. Furthermore, CHA greatly enhanced the bactericidal effect of gentamicin on viable bacteria in the biofilms. These results suggest that CHA, acting as a QSI, may enhance the susceptibility of *P. aeruginosa* to gentamicin by disrupting its QS-regulated biofilms. Thus, combining CHA with gentamicin presents a promising alternative to current single-drug therapies. Our work would provide a new strategy for the prevention and treatment of *P. aeruginosa* in food, drinking water, public health, nosocomial infection and many other aspects. Unfortunately, for the clinical application of CHA and MCHA, much work remains to be performed in further study, such as their cytotoxicity in eukaryotic cells and the efficacy *in vivo*.

## 5 Conclusions

This study is the first to report the inhibitory effects of CHA and MCHA against the QS system in *P. aeruginosa*. Results showed CHA and MCHA inhibited the production of multiple virulence factors, including pyocyanin, proteases, rhamnolipid, and siderophore, and suppressed biofilm formation and swimming and swarming motilities in *P. aeruginosa.* Furthermore, CHA exhibited potent synergistic inhibitory effects with gentamicin on biofilm formation and biofilm cell dispersal. These results indicate that CHA and MCHA, as two novel QSIs, offer promising potential for treating *P. aeruginosa* infections and reducing its virulence.

## Data availability statement

The original contributions presented in the study are included in the article/**Supplementary Material**. Further inquiries can be directed to the corresponding author.

## Ethics statement

The manuscript presents research on animals that do not require ethical approval for their study.

## Author contributions

DP: Data curation, Formal analysis, Investigation, Methodology, Software, Visualization, Writing – original draft, Writing – review & editing. HW: Investigation, Methodology, Software, Writing – review & editing. JL: Writing – original draft, Writing – review & editing. BW: Funding acquisition, Supervision, Writing – review & editing. AJ: Conceptualization, Funding acquisition, Investigation, Project administration, Resources, Supervision, Validation, Writing – review & editing.
