# Supplementary material for: Two cinnamoyl hydroxamates as potential quorum sensing inhibitors against Pseudomonas aeruginosa
Source: Front Cell Infect Microbiol. 2024 Aug 6;14:1424038. doi: 10.3389/fcimb.2024.1424038 (PMC11333444; doi:10.3389/fcimb.2024.1424038)
Supplement: Supplementary file 1 [file DataSheet_1.docx]

**Two cinnamoyl hydroxamates are potential quorum sensing inhibitors against *Pseudomonas aeruginosa***

Deng Pan ^a, b, #^, Hua Wu ^a^, Jun-Jian Li ^b, #^, Bo Wang ^a,^ *, Ai-Qun Jia ^a,^ *

^a^ Hainan General Hospital, Hainan Affiliated Hospital of Hainan Medical University, Haikou 570311, China.

^b^ Key Laboratory of Tropical Biological Resources of Ministry of Education, School of Pharmaceutical Sciences, Hainan University, Haikou 570228, China.

*** Corresponding author**: Ai-Qun Jia, [aqjia@hainmc.edu.cn](mailto:ajia@hainanu.edu.cn).

Postal address: Hainan General Hospital, Central Laboratory, 19 Xiuhua Road, Haikou 570311, Hainan, China

^#^ The author contributed to this work equally.

**Synthesis of seven cinnamoyl hydroxamates**

Cinnamic acid or its derivatives, *N*, *N*'-carbonyldiimidazole (CDI) and hydroxylamine hydrochloride were procured from Macklin Biochemical (Shanghai, China). The solvents were purified and dried by standard procedures prior to use. Reactions were performed under an argon atmosphere. The chemical shifts values (δ) in the NMR spectra were calculated in parts per million (ppm) relative to tetramethylsilane (TMS) using residual DMSO signal as internal standard. The synthesis procedures are referenced to a reported methods (1). These procedures involved the addition of CDI (4.5 mmol, 1.5 eq) to a solution of cinnamic acid or its derivatives (3.0 mmol) in anhydrous tetrahydrofuran (THF, 5 mL), followed by stirring for 1 h. 2M aqueous hydroxylamine hydrochloride (3 mL, 6 mmol) was then added to the reaction mixture and further stirred until the completion of the reaction, as monitored by thin-layer chromatography (TLC). After completion, the reaction mixture was diluted with 5% aq. KHSO_4_ (30 mL) and extracted with EtOAc (2 × 30 mL). The combined organic phase was washed with brine (30 mL) and dried over Na_2_SO_4_. The extract was then subjected to vacuum filtration and concentration to obtain the crude products, then submit to column chromatography to purify them.

**The identification of seven cinnamoyl hydroxamates**

**Cinnamohydroxamic acid (CHA, a)** White solid; ^1^H NMR (400 MHz, DMSO-d6) δ 10.78 (s, 1H), 9.07 (s, 1H), 7.56 (d, *J* = 6.9 Hz, 2H), 7.46 (d, *J* = 15.8 Hz, 1H), 7.43 – 7.32 (m, 3H), 6.47 (d, *J* = 15.8 Hz, 1H).

**3-Methoxy-cinnamohydroxamic acid (MCHA, b)** White solid; ^1^H NMR (400 MHz, DMSO-d6) δ 10.80 (s, 1H), 9.12 (s, 1H), 7.45 (d, *J* = 15.6 Hz, 1H), 7.31 (t, *J* = 7.6 Hz, 1H), 7.13 (d, *J* = 11.8 Hz, 2H), 6.93 (d, *J* = 7.4 Hz, 1H), 6.49 (d, *J* = 15.6 Hz, 1H), 3.77 (s, 3H).

**4-Methoxy-cinnamohydroxamic acid (c)** Off-white solid; ^1^H NMR (400 MHz, DMSO-d6) δ 10.68 (s, 1H), 8.99 (s, 1H), 7.51 (d, *J* = 8.6 Hz, 2H), 7.40 (d, *J* = 15.8 Hz, 1H), 6.97 (d, *J* = 8.7 Hz, 2H), 6.32 (d, *J* = 15.8 Hz, 1H), 3.78 (s, 3H).

**3,4-Dimethoxy-cinnamohydroxamic acid (d)** White solid; ^1^H NMR (400 MHz, DMSO-d6) δ 10.65 (s, 1H), 8.98 (s, 1H), 7.40 (d, *J* = 15.7 Hz, 1H), 7.18 – 7.08 (m, 2H), 6.98 (d, *J* = 8.3 Hz, 1H), 6.35 (d, *J* = 15.8 Hz, 1H), 3.80 (s, 3H), 3.79 (s, 3H).

**4-Hydroxyl-cinnamohydroxamic acid (e)** Dark grey solid ^1^H NMR (400 MHz, DMSO-d6) δ 10.66 (s, 1H), 9.89 (s, 1H), 8.97 (s, 1H), 7.40 (s, 1H), 7.36 (d, *J* = 16.4 Hz, 2H), 6.79 (d, *J* = 8.5 Hz, 2H), 6.26 (d, *J* = 15.7 Hz, 1H).

**4-Fluoro-cinnamohydroxamic acid (f)** Yellow solid; ^1^H NMR (400 MHz, DMSO-d6) δ 10.76 (s, 1H), 9.05 (s, 1H), 7.63 (dd, *J* = 8.0, 5.7 Hz, 2H), 7.46 (d, *J* = 15.8 Hz, 1H), 7.25 (t, *J* = 8.7 Hz, 2H), 6.41 (d, *J* = 15.8 Hz, 1H).

**3,4-Difluoro-cinnamohydroxamic acid (g)** Off-white solid; ^1^H NMR (400 MHz, DMSO-d6) δ 10.83 (s, 1H), 9.11 (s, 1H), 7.69 (dd, *J* = 10.7, 8.3 Hz, 1H), 7.51 – 7.45 (m, 2H), 7.43 (d, *J* = 11.2 Hz, 1H), 6.45 (d, *J* = 15.8 Hz, 1H).

**The determination of QS inhibitory activities of these seven cinnamoyl hydroxamates**


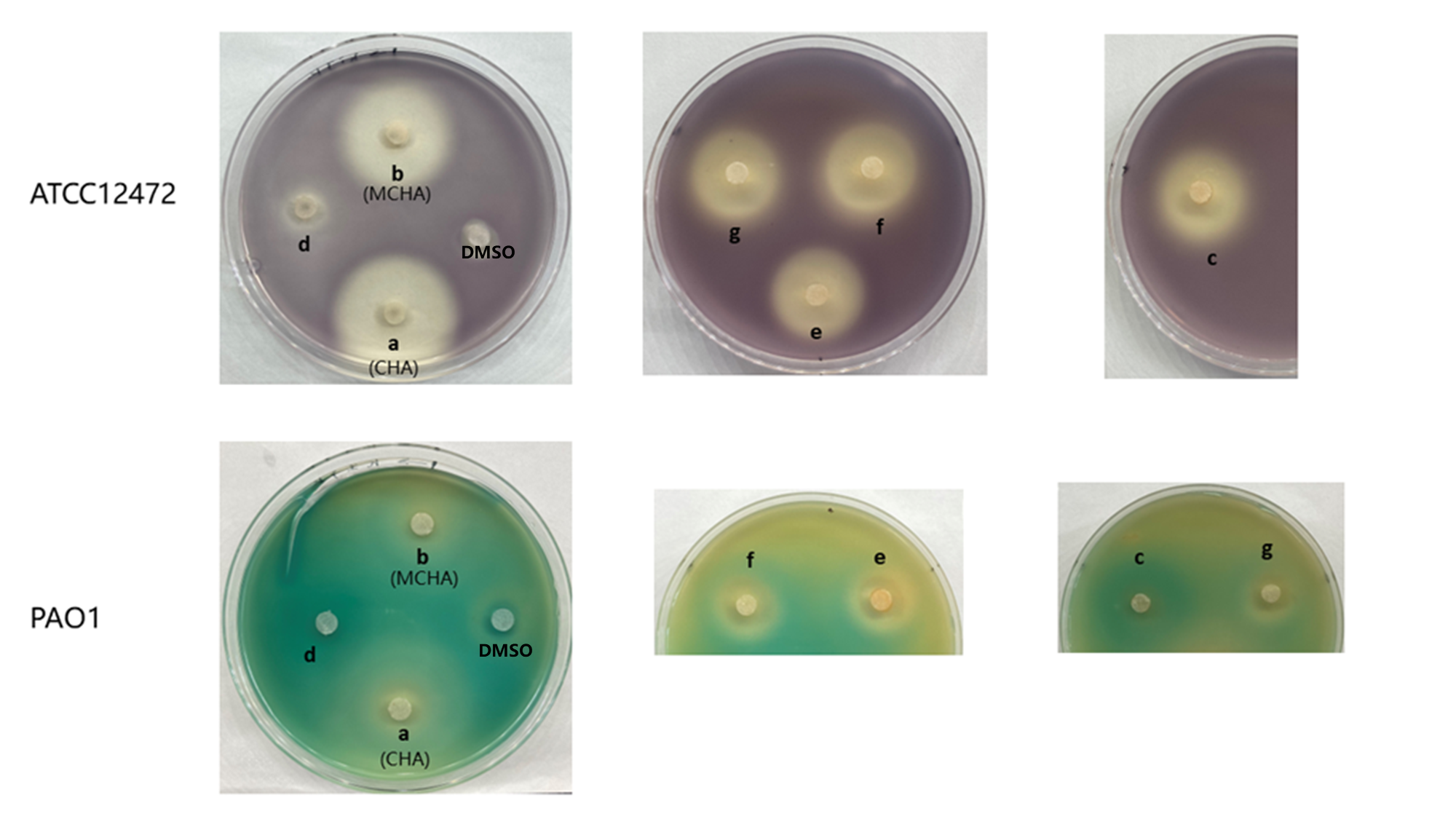


Table S1. PCR primers for RT-qPCR

| Genes | Primer direction | Sequences (5’-3’) | Amplicon sizes (bp) |
| --- | --- | --- | --- |
| *lasR* | Forward | CCTGTGGATGCTCAAGGACTA | 173 |
|  | Reverse | CATTGGCTTCCGAGCAGTT |  |
| *rhlR* | Forward | CTCCTCGGAAATGGTGGTCT | 138 |
|  | Reverse | GAAAGCACGCTGAGCAAATT |  |
| *rhlI* | Forward | GAAGATATTCTGGTCCAGCCTG | 147 |
|  | Reverse | TCGCCCTTGACCTTCTGC |  |
| *pqsH* | Forward | GAATTCACCAAGGCAGGCA | 85 |
|  | Reverse | GAACCGTCGTGGGCGTAT |  |
| *lasA* | Forward | TGAATGACGACCTGTTCCTCTAC | 152 |
|  | Reverse | GGTCAGCAACACTTTCGGG |  |
| *lasB* | Forward | AAGACCGAGAATGACAAAGTGG | 92 |
|  | Reverse | CCGGGAATCAGGTAGGAGAC |  |
| *rhlA* | Forward | ACGAGACCGTCGGCAAATA | 153 |
|  | Reverse | GCTCCAGGCAAGCCAAGT |  |
| *rhlB* | Forward | GTGCTGCTGCCCTGTGC | 193 |
|  | Reverse | AAGCGATACTGTGCGGTTGT |  |
| *phzM* | Forward | TGTTCTACGGCGAGGAGTTC | 180 |
|  | Reverse | TCTCGTGGAATGCCAGGTT |  |
| *oprL* | Forward | GACCCGAACGCAGGCTAT | 154 |
|  | Reverse | CTTTCGCGTGTACGTCCAG |  |
| *pslA* | Forward | GCAAGCTGGTGATCTTCTGG | 188 |
|  | Reverse | GGAGCGGATGTCGTGGTT |  |
| *flhF* | Forward | AGCGCAAGGACCGTCAAT | 127 |
|  | Reverse | AGTTCGAAACGCATTGCCT |  |
| *motD* | Forward | TATGCCGACTTCATCACCCT | 96 |
|  | Reverse | GGTCTCCGAGAGGATCTTGTAC |  |
| *rpsL* | Forward | AGGTTTCCTCGTACATCGGTG | 132 |
|  | Reverse | CCGAGGTGTCCAGCGAAC |  |

Note: All primers were designed by Primer Premier 5.0 software (PREMIER Biosoft, San Francisco, USA) and synthesized by Sangon Biotech (Shanghai, China).

Table S2. MICs of CHA, MCHA and antibiotics

| Chemicals | MICs (μg/mL) | FICIs |
| --- | --- | --- |
|  | PAO1 | CHA |
| CHA | 600 | - |
| MCHA | 800 | - |
| gentamicin | 4 | 0.583 |
| polymyxin B | 4 | 0.833 |
| aztreonam | 4 | 1.667 |


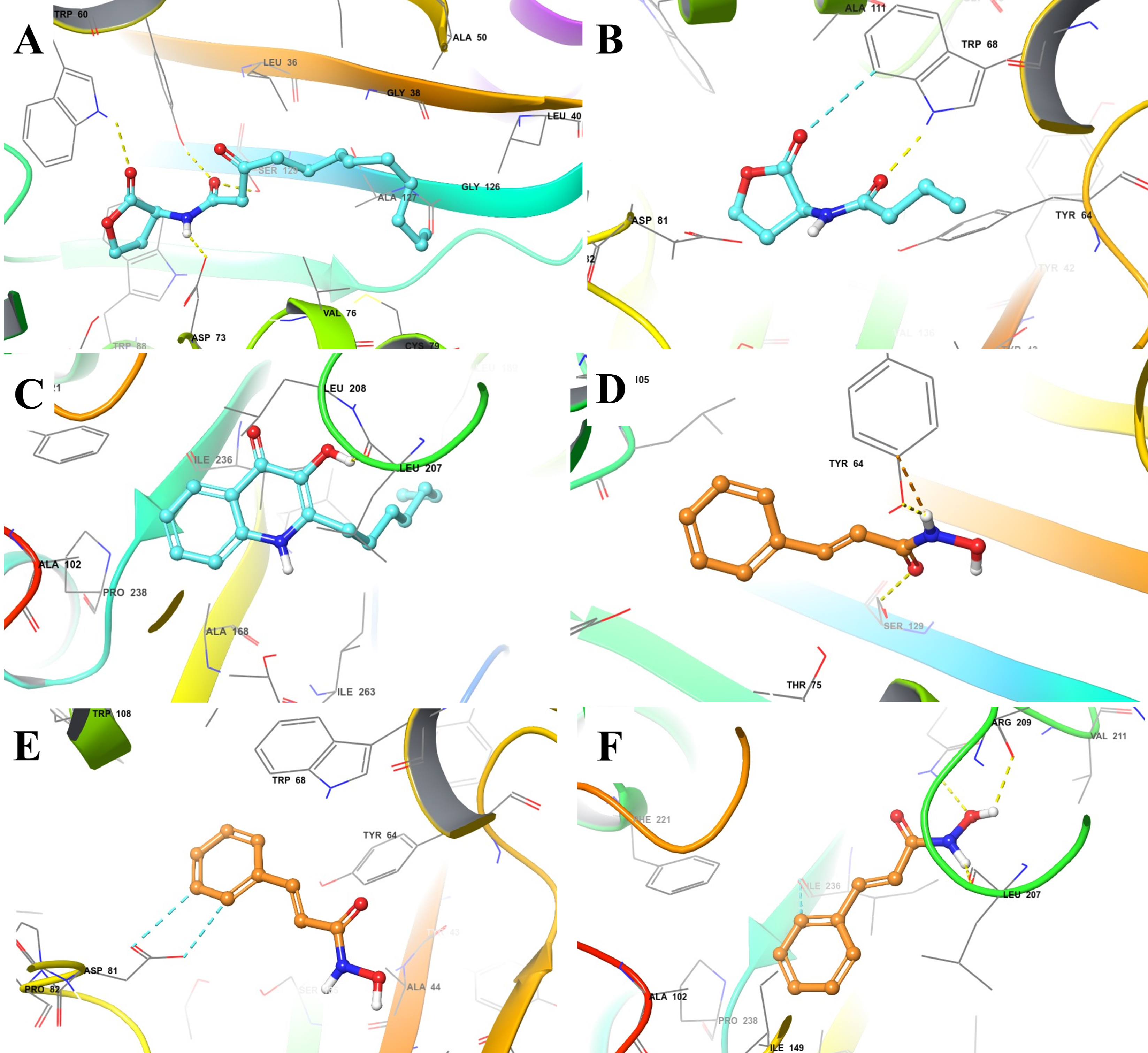


1. N. Usachova, G. Leitis, A. Jirgensons, I. Kalvinsh, Synthesis of Hydroxamic Acids by Activation of Carboxylic Acids with *N*,*N′*-Carbonyldiimidazole: Exploring the Efficiency of the Method. *Synthetic Communications* **40**, 927-935 (2010).

**The significance P value and the degree of freedom (df) obtained from statistical analyses on the result of each experiment**

The experiment of CHA on *P. aeruginosa* PAO1 biofilm disruption

| Dunnett's multiple comparisons test | Adjusted P Value | df |
| --- | --- | --- |
| DMSO vs. 50 | 0.0003 | 4 |
| DMSO vs. 100 | <0.0001 |  |
| DMSO vs. 200 | <0.0001 |  |

The experiment of MCHA on *P. aeruginosa* PAO1 biofilm disruption

| Dunnett's multiple comparisons test | Adjusted P Value | df |
| --- | --- | --- |
| DMSO vs. 75 | <0.0001 | 3 |
| DMSO vs. 150 | <0.0001 |  |
| DMSO vs. 300 | <0.0001 |  |

The inhibition experiment of CHA on pyocyanin of *P. aeruginosa* PAO1

| Dunnett's multiple comparisons test | Adjusted P Value | df |
| --- | --- | --- |
| DMSO vs. Hor | <0.0001 | 4 |
| DMSO vs. 50 | <0.0001 |  |
| DMSO vs. 100 | <0.0001 |  |
| DMSO vs. 200 | <0.0001 |  |

The inhibition experiment of MCHA on pyocyanin of *P. aeruginosa* PAO1

| Dunnett's multiple comparisons test | Adjusted P Value | df |
| --- | --- | --- |
| DMSO vs. Hor | <0.0001 | 4 |
| DMSO vs. 75 | <0.0001 |  |
| DMSO vs. 150 | <0.0001 |  |
| DMSO vs. 300 | <0.0001 |  |

The inhibition experiment of CHA on elastase of *P. aeruginosa* PAO1

| Dunnett's multiple comparisons test | Adjusted P Value | df |
| --- | --- | --- |
| DMSO vs. Hor | 0.0037 | 4 |
| DMSO vs. 50 | 0.0006 |  |
| DMSO vs. 100 | <0.0001 |  |
| DMSO vs. 200 | <0.0001 |  |

The inhibition experiment of MCHA on elastase of *P. aeruginosa* PAO1

| Dunnett's multiple comparisons test | Adjusted P Value | df |
| --- | --- | --- |
| DMSO vs. Hor | 0.0011 | 4 |
| DMSO vs. 75 | 0.0007 |  |
| DMSO vs. 150 | <0.0001 |  |
| DMSO vs. 300 | <0.0001 |  |

The inhibition experiment of CHA on protease of *P. aeruginosa* PAO1

| Dunnett's multiple comparisons test | Adjusted P Value | df |
| --- | --- | --- |
| DMSO vs. Hor | <0.0001 | 4 |
| DMSO vs. 50 | 0.0023 |  |
| DMSO vs. 100 | <0.0001 |  |
| DMSO vs. 200 | <0.0001 |  |

The inhibition experiment of MCHA on protease of *P. aeruginosa* PAO1

| Dunnett's multiple comparisons test | Adjusted P Value | df |
| --- | --- | --- |
| DMSO vs. Hor | <0.0001 | 4 |
| DMSO vs. 75 | 0.0952 |  |
| DMSO vs. 150 | <0.0001 |  |
| DMSO vs. 300 | <0.0001 |  |

The inhibition experiment of CHA on rhamnolipid of *P. aeruginosa* PAO1

| Dunnett's multiple comparisons test | Adjusted P Value | df |
| --- | --- | --- |
| DMSO vs. Hor | <0.0001 | 4 |
| DMSO vs. 50 | 0.0186 |  |
| DMSO vs. 100 | 0.0007 |  |
| DMSO vs. 200 | <0.0001 |  |

The inhibition experiment of MCHA on rhamnolipid of *P. aeruginosa* PAO1

| Dunnett's multiple comparisons test | Adjusted P Value | df |
| --- | --- | --- |
| DMSO vs. Hor | 0.0011 | 4 |
| DMSO vs. 75 | 0.0007 |  |
| DMSO vs. 150 | <0.0001 |  |
| DMSO vs. 300 | <0.0001 |  |

The inhibition experiment of CHA on siderophore of *P. aeruginosa* PAO1

| Dunnett's multiple comparisons test | Adjusted P Value | df |
| --- | --- | --- |
| DMSO vs. Hor | 0.0006 | 4 |
| DMSO vs. 50 | 0.0009 |  |
| DMSO vs. 100 | <0.0001 |  |
| DMSO vs. 200 | <0.0001 |  |

The inhibition experiment of MCHA on siderophore of *P. aeruginosa* PAO1

| Dunnett's multiple comparisons test | Adjusted P Value | df |
| --- | --- | --- |
| DMSO vs. Hor | 0.0003 | 4 |
| DMSO vs. 75 | <0.0001 |  |
| DMSO vs. 150 | <0.0001 |  |
| DMSO vs. 300 | <0.0001 |  |

The inhibition experiment of CHA on QS-related gene expression in *P. aeruginosa* PAO1

| t-test | P Value | df |
| --- | --- | --- |
| DMSO vs. lasR | 0.0014 | 4 |
| DMSO vs. rhlR | 0.0024 |  |
| DMSO vs. rhlI | <0.0001 |  |
| DMSO vs. pqsH | 0.0029 |  |
| DMSO vs. lasA | 0.0051 |  |
| DMSO vs. lasB | <0.0001 |  |
| DMSO vs. rhlA | 0.0063 |  |
| DMSO vs. rhlB | 0.0033 |  |
| DMSO vs. phzM | 0.0029 |  |
| DMSO vs. oprL | <0.0001 |  |
| DMSO vs. pslA | 0.0096 |  |
| DMSO vs. flhF | 0.0309 |  |
| DMSO vs. motD | 0.2659 |  |

The inhibition experiment of MCHA on QS-related gene expression in *P. aeruginosa* PAO1

| t-test | P Value | df |
| --- | --- | --- |
| DMSO vs. lasR | <0.0001 | 4 |
| DMSO vs. rhlR | 0.0118 |  |
| DMSO vs. rhlI | 0.0011 |  |
| DMSO vs. pqsH | 0.0111 |  |
| DMSO vs. lasA | 0.0005 |  |
| DMSO vs. lasB | <0.0001 |  |
| DMSO vs. rhlA | 0.0075 |  |
| DMSO vs. rhlB | 0.0031 |  |
| DMSO vs. phzM | 0.0001 |  |
| DMSO vs. oprL | <0.0001 |  |
| DMSO vs. pslA | 0.1091 |  |
| DMSO vs. flhF | 0.1744 |  |
| DMSO vs. motD | 0.3764 |  |

The CFU analysis experiment of synergistic anti-biofilm activity of CHA in combination with gentamicin

| Dunnett's multiple comparisons test | Adjusted P Value | df |
| --- | --- | --- |
| DMSO vs. GEN+CHA | <0.0001 | 3 |
| GEN vs. GEN+CHA | <0.0001 |  |
| CHA vs. GEN+CHA | <0.0001 |  |

The crystal violet staining experiment of synergistic anti-biofilm activity of CHA in combination with gentamicin

| Dunnett's multiple comparisons test | Adjusted P Value | df |
| --- | --- | --- |
| DMSO vs. GEN+CHA | <0.0001 | 3 |
| GEN vs. GEN+CHA | <0.0001 |  |
| CHA vs. GEN+CHA | <0.0001 |  |
